# Exosome-Derived miR-11987 in Bovine Milk Inhibits Obesity Through Browning of White Fat

**DOI:** 10.3390/ijms26136006

**Published:** 2025-06-23

**Authors:** In-Seon Bae, Sang Hoon Kim

**Affiliations:** 1 Department of Animal Biotechnology and Environment, National Institute of Animal Science, Iseo-myeon, Wanju-gun 55365, Jeonbuk-do, Republic of Korea; 2 Department of Biology, College of Sciences, Kyung Hee University, Seoul 02447, Republic of Korea

**Keywords:** milk, exosome, microRNA, obesity, miR-11987

## Abstract

The global obese population accounts for approximately 30% of the total population and continues to increase. White adipocytes, which accumulate in the body for energy storage, are associated with obesity. Mechanisms that activate browning of white adipocytes are an attractive therapeutic target for obesity and metabolic disorders. Exosomes are nano-sized biovesicles that play a role in cell-to-cell communication though the transfer of cargos such as microRNAs. Although milk exosomes contain many endogenous microRNA molecules, the role of microRNAs in milk exosomes is limited. Therefore, the aim of this study was to investigate the effects of milk exosomes on the browning of white adipocyte. Mouse pre-adipocytes (3T3-L1) and human adipose-derived stem cells (hADSCs) were differentiated and exposed to milk exosomes. Compared to control, milk exosomes promoted the expression of thermogenic genes and cellular mitochondrial energy metabolism in both 3T3-L1 cells and hADSCs. Additionally, milk exosomes were orally administered to mice fed a high-fat diet. As the intake of milk exosomes increased, the mice’s body weight decreased. Milk exosomes also increased the protein levels of thermogenic genes and mitochondrial-related genes in mouse adipose tissue. The overexpression of miR-11987, which is abundant in milk exosomes, in both 3T3-L1 cells and hADSCs led to the increased expression of thermogenic genes and mitochondrial activity. Our results support that bovine-specific miR-11987 in milk exosomes promotes the browning of white adipocytes. Therefore, milk exosome and milk exosomal miR-11987 could have significant clinical implications for obesity and metabolic syndrome.

## 1. Introduction

Obesity is a worldwide health problem, and the cause of obesity is an energy imbalance between calories consumed and calories expended, leading to excess fat in the body [1,2]. Increased body fat, especially visceral fat, is associated with a high risk of metabolic disease [3]. The global obese population is about 2.1 billion people, accounting for approximately 30% of the total population. The obese population continues to increase, which has a significant impact on health impairment and reduced quality of life. In particular, the global prevalence of obesity causes complications such as cardiovascular diseases, type 2 diabetes, and cancer.

White adipocytes store excess energy in the form of triglycerides and play a major role in energy homeostatic control. These cells not only act as a reservoir for energy storage but also secrete paracrine factors like adipokines to regulate other metabolic tissues [4]. However, the excessive accumulation of white adipocytes in the body causes health complications. In contrast, brown adipocytes maintain body temperature and increase energy consumption through thermogenesis via the activation of uncoupling protein 1 (UCP1), which uncouples the cellular respiratory chain and dissipates the proton electrochemical gradient generated by oxidative phosphorylation in the form of heat [5]. White adipocytes contain a single, large lipid droplet, whereas brown adipocytes contain multilocular lipid droplets and numerous mitochondria. Beige adipocytes, similar to brown adipocytes, express a high level of UCP1 and emerge within white adipose tissue through multiple stimuli, such as cold exposure, spicy food, and the activation of β-adrenergic receptors, thereby enhancing whole-body energy expenditure [6]. These thermogenic adipocytes are negatively associated with obesity and metabolic dysfunction, such as insulin resistance and inflammation [7].

MicroRNAs are small non-coding RNAs, which play a role in gene regulation in biological processes. These small molecules bind to the 3’untranslated region (3’UTR) of mRNA to post-transcriptionally inhibit gene expression, either by inhibiting target gene translation or degrading the targeted transcript. In adipocytes, several microRNAs are required for brown adipogenesis. White adipocytes are converted into beige adipocytes by miR-26a and miR-26b [8]. MiR-196a induces the browning of white adipocytes via targeting HOXC8, a repressor of C/EBPβ, an essential regulator of brown and beige fat development [9]. Moreover, miR-455 is found to promote the differentiation of brown adipocytes by targeting RUNX1T1 and Necidin, key adipogenic repressors [10]. In contrast, miR-133 and miR-27 inhibit the differentiation of brown adipocytes through Prdm16, a key regulator of the adipocyte browning [11,12].

Exosomes are small membrane-encapsulated vesicles with diameters ranging from 50 nm to 200 nm. These vesicles are present in biological fluids, including blood, urine, saliva, and breast milk. Exosomes contain bioactive ingredients such as mRNA, protein, and microRNA. Exosomes derived from foods such as milk, grape, ginger, and broccoli are absorbed into the digestive tract [13,14,15,16]. After being absorbed into the body, food-derived exosomes also affect the function of recipient cells.

Cow milk has been used for thousands of years as a source of human nutrition. Milk contains carbohydrates, proteins, fat vitamins, minerals, and bioactive compounds, which provides energy and promotes metabolism. Cohort studies have reported that milk suppresses obesity [17,18,19]. According to the National Health and Nutrition Examination Survey (NHANES), overweight or obese people aged 2 to 20 years consume less milk than healthy weight people [18]. Another cohort study investigated the association between milk consumption and obesity in 3-year-old Latinos. Of the 145 people surveyed, 17% were obese, and they were found to consume less milk [19]. Through this cohort study, it can be assumed that the consumption of milk can suppress obesity. As a component in milk, calcium is known to suppress obesity. Calcium-rich milk reduces body weight and improves insulin sensitivity [20]. In addition, caseins and whey proteins reduce body fat and increase muscle mass to suppress obesity [21,22]. According to the protein analysis of milk fat globules, AIFM2 and ACSL1 proteins are known to induce brown adipocyte differentiation [23,24]. Although milk intake reduces obesity, studies on microRNAs derived from milk involved in obesity are limited.

In this study, we explored the milk exosomes containing microRNAs that inhibit obesity by inducing the browning of white fat. Our findings highlight the therapeutic potential of milk exosome-derived microRNAs in the treatment of obesity.

## 2. Results

### 2.1. Characterization of Bovine Milk Exosomes

**Figure 1:**
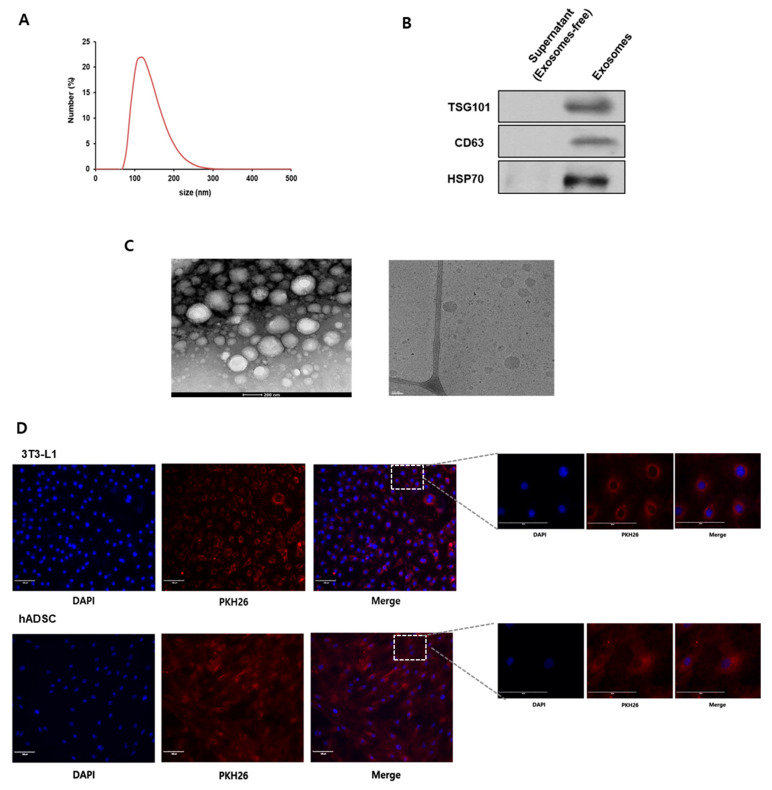
Characterization of milk exosomes. (**A**) Quantification of the concentrations of various-sized particles in the extracted milk exosomes through Nanoparticle Tracking Analysis. (**B**) The expression levels of exosome markers CD63, TSG101, and HSP70 in the isolated milk exosomes were detected by Western blot. (**C**) Cryo-transmission electron microscopy (scale bar, 50 nm) depicted milk exosome morphology. (**D**) 3T3-L1 cells and hADSCs were analyzed by fluorescence microscopy after incubation with exosomes labeled with fluorescent marker PKH26. Nuclei were stained with 4′,6-diamidino-2-phenylindole (DAPI; blue). Scale bar, 100 μm.

To investigate the characterization of the milk exosomes, the size distribution of exosomes was observed by dynamic light scattering. As a result, milk exosomes were approximately 80~220 nm in diameter (Figure 1A). We confirmed that the isolated exosomes expressed exosome-specific markers, such as TSG101, CD63, and HSP70 by Western blot (Figure 1B). Under electron microscopy analysis, exosomes were found to have a round shape within a 200 nm diameter (Figure 1C). Next, cells were treated with fluorescent dye PKH26-labeled exosomes to verify the exosome uptake into cells. As a result, we observed that PKH26-labeled exosomes were observed in 3T3-L1 cells and human adipose-derived stem cells (hADSCs) (Figure 1D).

### 2.2. Milk Exosomes Induce Browning of White Adipocytes

**Figure 2:**
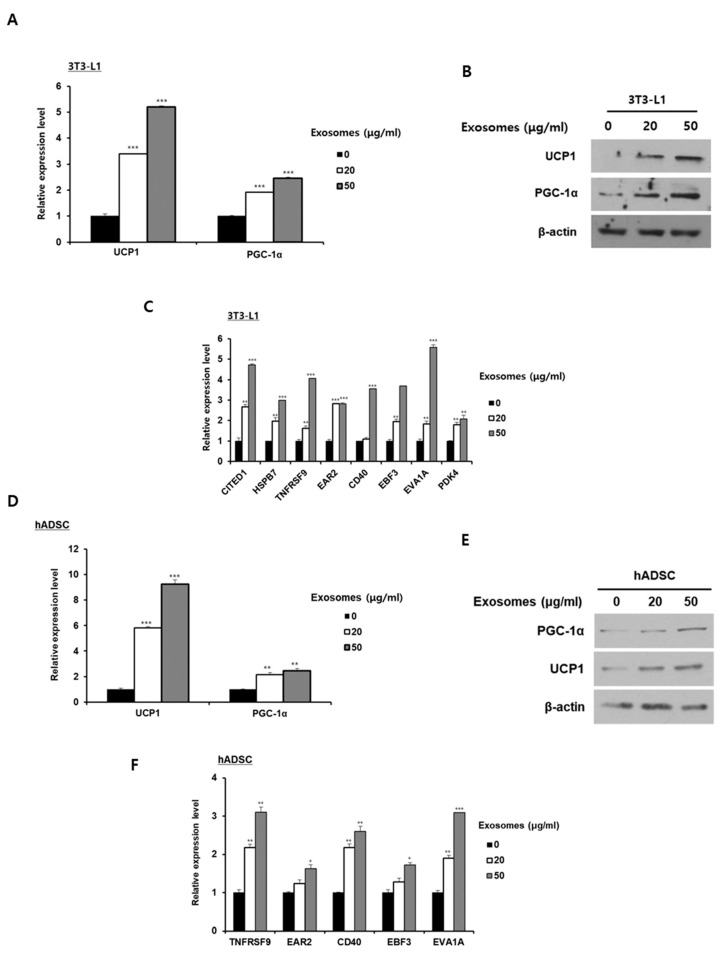
Milk exosomes induce the browning of white adipocytes in differentiated mature adipocytes in mice and humans. (**A**) The mRNA expression levels of thermogenic genes PGC-1α and UCP1 were measured in 3T3-L1 cells treated with or without milk exosomes. *** *p* < 0.001. (**B**) PGC-1α and UCP1 protein expression levels in 3T3-L1 cells treated with milk exosomes were detected by Western blot. (**C**) Beige adipocyte-specific gene expression was assessed in milk exosome-treated 3T3-L1 cells by qRT-PCR. ** *p* < 0.01; *** *p* < 0.001. (**D**) Thermogenic gene expression levels were determined in the hADSCs exposed to milk exosomes ** *p* < 0.01. *** *p* < 0.001. (**E**) PGC-1α and UCP1 protein levels in milk exosome-treated hADSCs were examined by Western blot. (**F**) Levels of beige adipocyte-specific mRNAs in hADSCs treated with milk exosomes were measured by qRT-PCR. * *p* < 0.05; ** *p* < 0.01; *** *p* < 0.001.

To investigate the effect of milk exosomes on the browning of white adipocytes, the expression levels of thermogenic genes were measured in 3T3-L1 cells and hADSCs. The mRNA and protein expression levels for thermogenic genes UCP1 and PGC-1α were significantly higher in mature 3T3-L1 adipocytes exposed to 20 or 50 μg/mL of milk exosomes than in control cells (Figure 2A,B). Furthermore, the expression of beige adipocyte-specific markers CITED1, HSPB7, TNFRSF9, EAR2, CD40, EBF3, EVA1A, and PDK4 mRNA in 3T3-L1 cells treated with milk exosomes increased in a dose-dependent manner (Figure 2C). When hADSCs were treated with milk exosomes at a concentration of 20 or 50 μg/mL, the mRNA and protein expression levels of thermogenic genes gradually increased (Figure 2D,E). Likewise, the mRNA expression of genes encoding beige adipocyte-specific markers in hADSCs was gradually upregulated as the treatment of milk exosomes increased (Figure 2F). These results indicate that milk exosomes can induce the browning of 3T3-L1 adipocytes and hADSCs by increasing the expression of beige adipocyte-related genes.

### 2.3. Milk Exosomes Improve Obesity in Mice Through Thermogenic Activation

**Figure 3:**
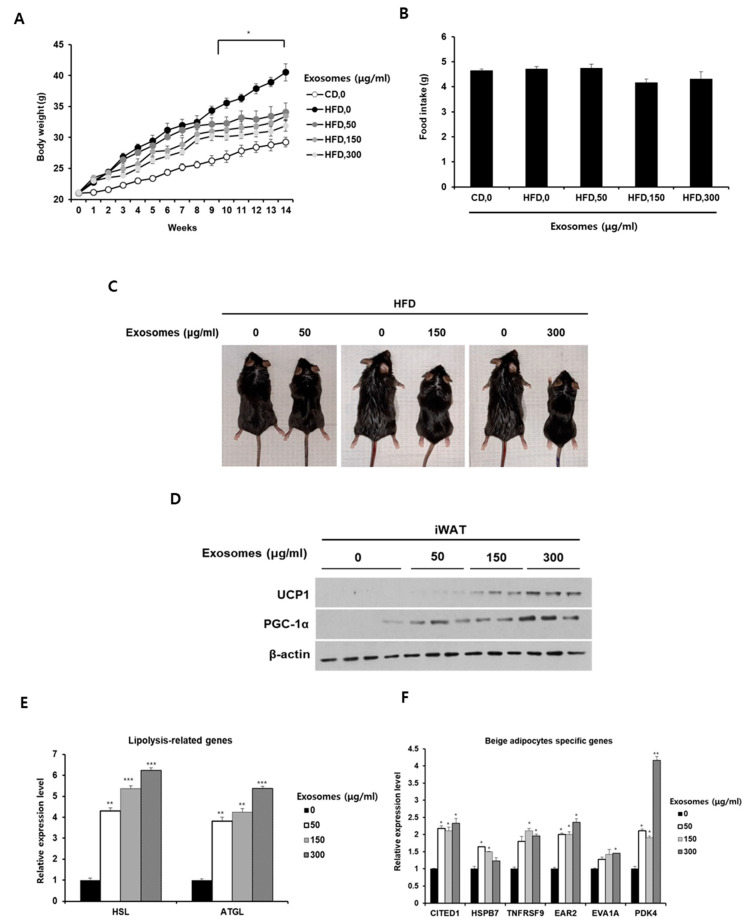
Milk exosomes induce the expression of thermogenic genes in obese mice. Eight-week-old C57BL6/J wild-type male mice were orally administered milk exosomes (50, 100, or 300 μg/mL) once every three days for fourteen weeks. (**A**) Body weight was measured during the experimental period. Statistics were calculated by comparing the weight of high-fat diet (HFD)-fed mice supplemented with milk exosomes and that of HFD-fed mice with PBS. * *p* < 0.05. (**B**) Food intake was monitored and measured according to the experimental protocol. (**C**) Representative images of HFD-fed animals. (**D**) The protein levels of UCP1 and PGC-1α in inguinal adipose tissue (iWAT) of mice fed HFD supplemented with milk exosomes were measured by Western blot. (**E**) Total RNA was isolated from mouse iWAT, and mRNA expression was analyzed for HSL and ATGL by qRT-PCR. ** *p* < 0.01; *** *p* < 0.001. (**F**) Total RNA was extracted from mouse iWAT, and the levels of beige adipocyte-specific markers were analyzed by qRT-PCR. * *p* < 0.05; ** *p* < 0.01.

Given that milk exosomes induced the browning of white adipocytes in mouse and human cells, we next determined that milk exosomes have equivalent effects in mice. Mice fed either a normal chow diet or high-fat diets supplemented with 0, 50, 150, or 300 μg/mL milk exosomes for 14 weeks. As shown in Figure 3A, treatment with milk exosomes reduced body weight gain in mice fed a high-fat diet. Furthermore, as the intake of milk exosomes increased, the weight gain of mice decreased further (Figure 3A). Food intake in mice was not affected by the exosome treatments (Figure 3B). Mice fed a high-fat diet with milk exosomes appeared to be slimmer than mice fed a high-fat diet alone (Figure 3C). The protein level of thermogenic markers such as UCP1 and PGC1α was higher in adipose tissue derived from mice fed both a high-fat diet and milk exosomes than in adipose tissue derived from mice that did not receive milk exosomes (Figure 3D). As expected, the expressions of genes related to lipolysis and beige adipocyte-specific markers were increased in adipose tissue from milk exosome-treated mice (Figure 3E,F). These data demonstrate that thermogenic activation is increased in obese mice treated with milk exosomes.

### 2.4. Milk Exosomes Increase Mitochondrial Activity

**Figure 4:**
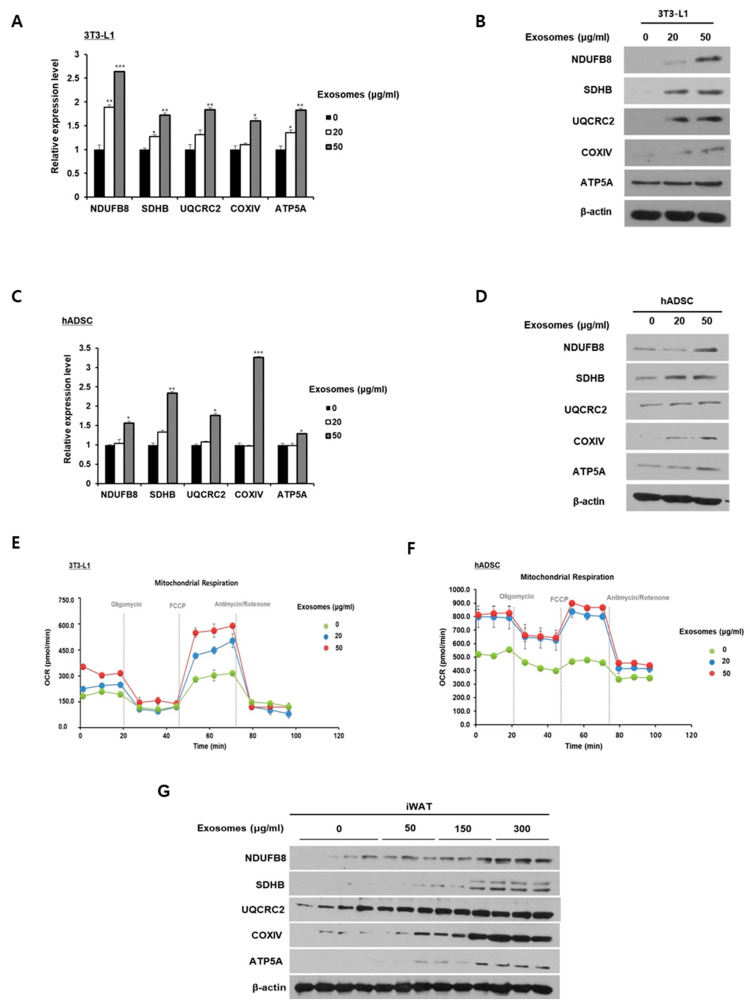
Milk exosomes promote mitochondrial respiration in adipocytes. Fully differentiated mature adipocytes from 3T3-L1 cells and hADSCs were treated with or without milk exosomes. (**A**) The mRNA levels of mitochondrial respiratory chain-related genes in milk exosome-treated 3T3-L1 cells were measured by qRT-PCR. * *p* < 0.05; ** *p* < 0.01; *** *p* < 0.001. (**B**) The protein expression levels of components in the mitochondrial electron transport chain complexes I-V were performed in 3T3-L1 supplemented with milk exosomes by Western blot. (**C**) The mRNA levels of mitochondrial oxidative phosphorylation (OXPHOS) genes (complexes I-V) were measured in hADSCs exposed to milk exosomes by qRT-PCR. * *p* < 0.05, ** *p* < 0.01, *** *p* < 0.001. (**D**) The protein levels of OXPHOS proteins (complexes I-V) measured in milk exosome-treated hADSCs. (**E**,**F**) Oligomycin, FCCP, and actinomycin/rotenone were added at the indicated points to determine uncoupled and maximal mitochondrial respiration, respectively. The effect of milk exosomes on the cellular oxygen consumption rate in 3T3-L1 cells or hADSCs was measured using an XF96 extracellular Flux Analyzer. (**G**) The levels of OXPHOX proteins in iWAT of mice fed both a high-fat diet and milk exosomes were assessed by Western blot.

To examine the effect of milk exosomes on mitochondrial activity in 3T3-L1 adipocytes and hADSCs, we investigated the expressions of subunit genes in mitochondrial electron transport complexes. The expressions of NDUFB8 (complex I), SDHB (complex II), UQCRC2 (complex III), COXIV (complex IV), and ATP5A (complex V) mRNAs were increased in milk exosome-treated cells compared with control cells (Figure 4A). At the protein level, the expression of these genes was upregulated in mature 3T3-L1 adipocytes as the concentration of milk exosomes increased (Figure 4B). Likewise, milk exosomes in hADSCs augmented the expression of mRNA-encoded subunits of the mitochondrial respiratory chain complexes (Figure 4C). The protein expression of these genes in hADSCs was also upregulated following treatment with milk exosomes (Figure 4D). Figure 4E,F show that milk exosomes prominently increased the mitochondrial oxygen consumption rate in 3T3-L1 adipocytes and hADSCs. The promoted oxygen consumption rate of milk exosome-treated 3T3-L1 adipocytes and hADSCs was observed after inserting an uncoupling agent, carbonyl cyanide 4-(trifluoromethoxy) phenylhydrazone (FCCP). In obese mice fed milk exosomes, the protein levels of the mitochondrial electron transport chain components NDUFB8, SDHB, UQCRC2, COXIV, and ATP5A were increased compared to control obese mice (Figure 4G). These results indicate that milk exosomes can promote mitochondrial activity in adipocytes.

### 2.5. MicroRNAs in Milk Exosomes Induce the Expression of Thermogenic Genes

**Figure 5:**
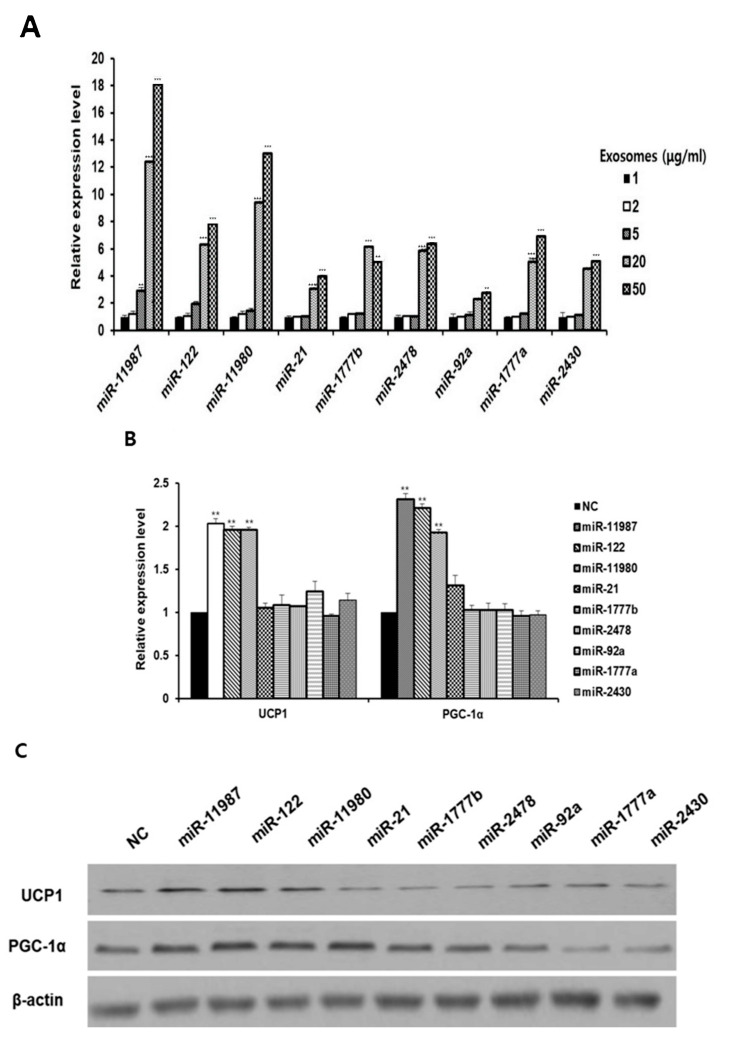
Milk exosomes contain several microRNAs. (**A**) The expression levels of microRNAs were measured by qRT-PCR in cells treated with milk exosomes. (**B**,**C**) 3T3-L1 cells were transfected with the milk exosomal microRNA mimic or negative control (NC). After inducing adipocyte differentiation, the expression of UCP1 and PGC-1α in cells was measured by qRT-PCR and Western blot. ** *p* < 0.01; *** *p* < 0.001.

RNA-seq was performed to find out the distribution of microRNA in milk exosomes. According to the results of RNA-seq analysis, nine microRNAs with read-count values of 2000 or higher were identified: miR-11987, miR-122, miR-11980, miR-21, miR-1777b, miR-2478, miR-92a, miR-1777a, and miR-2430 (Supplementary Table S2). Quantitative real-time PCR was conducted to confirm the expressions of selected microRNAs. As the milk exosome concentration increased, the expressions of these microRNAs in milk exosome-treated cells increased (Figure 5A). However, the levels of microRNA expressions did not match the read-count ranking of RNA-seq. We next determined that the selected nine microRNAs enhance the expression of thermogenic genes. In adipocytes transfected with miR-11987, miR-122, and miR-11980, the expression of UCP1 and PGC-1α mRNAs was increased (Figure 5B). We confirmed that miR-11987, miR-122, and miR-11980 upregulated the protein levels of these genes (Figure 5C). These results indicate that miR-11987, miR-122, and miR-11980, which are present in milk exosomes, promote the expression of thermogenic genes in adipocytes.

### 2.6. MiR-11987 Promotes Energy Expenditure in Adipocytes

**Figure 6:**
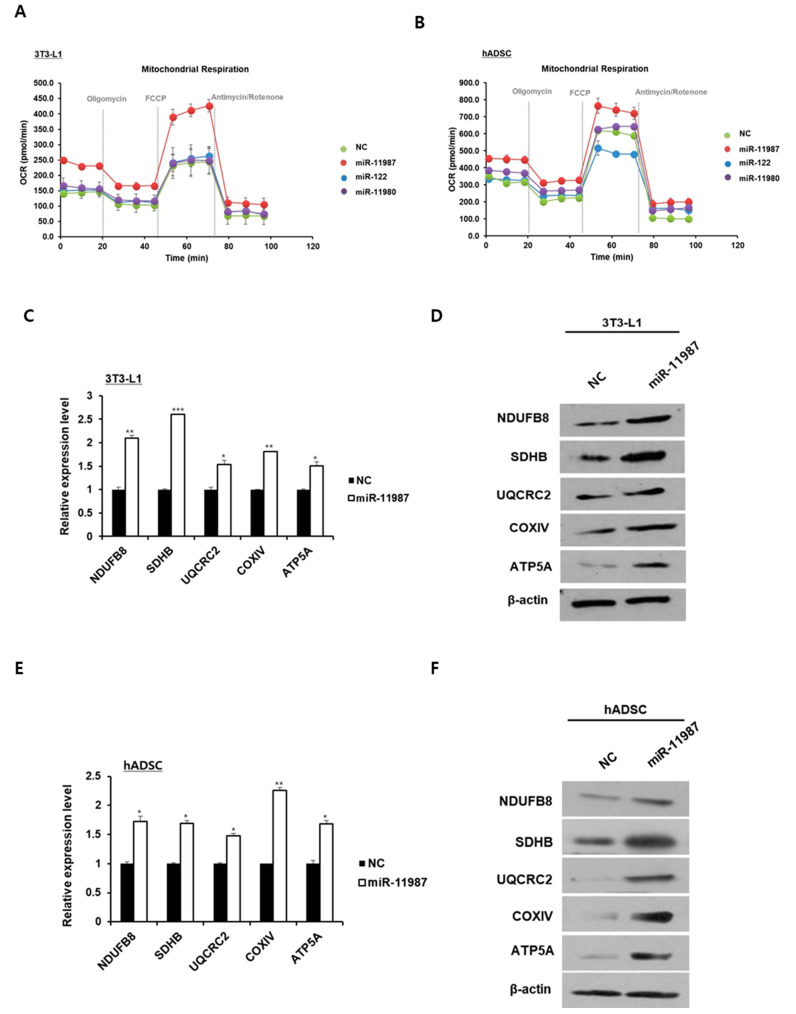
MiR-11987 promotes mitochondrial activity. miR-11987-, miR-122-, and miR-11980-transfected cells were differentiated into mature adipocytes in mouse and human cells. (**A**,**B**) Mitochondrial oxygen consumption was analyzed in 3T3-L1 cells and hADSCs. (**C**,**D**) The expression of genes encoding each subunit of the mitochondrial respiratory chain complexes in 3T3-L1 cells was evaluated by qRT-PCR and Western blot. * *p* < 0.05; ** *p* < 0.01; *** *p* < 0.001. (**E**,**F**) The levels of representative subunits of the respiratory complexes I–V in hADSCs were analyzed by qRT-PCR and Western blot. * *p* < 0.05; ** *p* < 0.001.

Next, we determined that miR-11987, miR-122, and miR-11980 stimulate mitochondrial activity. Oxygen consumption analysis demonstrated that miR-11987-transfected 3T3-L1 adipocytes displayed higher basal and maximal mitochondrial respiration than the negative control group, whereas miR-122- or miR-11980-transfected 3T3-L1 adipocytes did not display a change in mitochondrial respiration compared to the control group (Figure 6A). In hADSCs, oxygen consumption increased only in miR-11987 treatment (Figure 6B). Based on these data, we decided to choose miR-11987 for further study. In miR-11987-transfected 3T3-L1 adipocytes, mRNA analysis revealed an increment in the expressions of complex I subunit NDUFB8, complex II subunit SDHB, complex III subunit UQCRC2, complex IV subunit COXIV, and complex V subunit ATP5A (Figure 6C). Western blotting analysis showed increased levels of the mitochondrial electron transport chain complex I~V, NDUFB8, SDHB, UQCRC2, COXIV, and ATP5A proteins in miR-11987-transfected cells (Figure 6D). In hADSCs, we measured the levels of representative subunits of the respiratory complexes I-V. As shown in Figure 6E,F, the mRNA and protein levels of these subunits were similar to those of 3T3-L1 adipocytes. These results suggest that miR-11987 promotes mitochondrial activity in 3T3-L1 cells and hADSCs.

### 2.7. Milk Exosomes Increase Energy Expenditure Through Exosomal miR-11987

**Figure 7:**
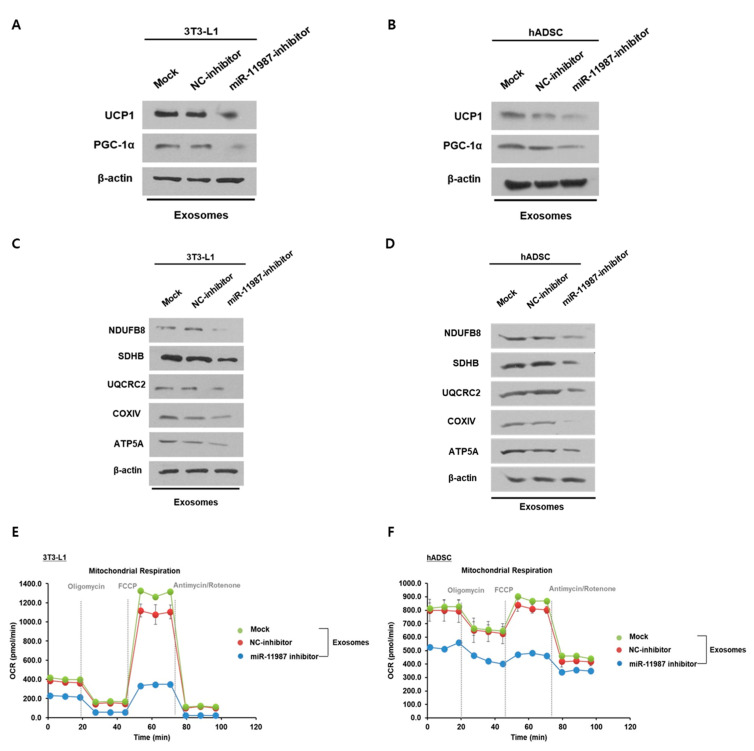
MiR-11987 induces the browning of white adipocytes. (**A**) The expression levels of thermogenic genes in 3T3-L1 cells treated with NC inhibitors or miR-11987 inhibitors were analyzed in the presence of milk exosomes (50 μg/mL). (**B**) The expression of UCP1 and PGC-1α was measured in NC inhibitor- or miR-11987 inhibitor-treated hADSCs supplemented with milk exosomes, as assessed by Western blot. (**C**) OXPHOS protein levels were examined in NC inhibitor- or miR-11987 inhibitor-treated 3T3-L1 cells supplemented with 50 μg/mL of exosomes. (**D**) The protein levels of OXPHOS genes in NC inhibitor- or miR-11987 inhibitor-treated hADSCs exposed to milk exosomes were measured by Western blot. (**E**,**F**) 3T3-L1 cells and hADSCs were analyzed with a Searhorse XF94 device to evaluate the mitochondrial oxygen consumption after miR-11987 knock-down.

To determine that miR-11987 in milk exosomes directly affects the browning of white adipocytes, 3T3-L1 cells and hADSCs exposed to milk exosomes were treated with miR-11987 inhibitors. 3T3-L1 cells treated with miR-11987 inhibitors in the presence of milk exosomes showed a lower expression level of thermogenic genes than that of control group (Figure 7A). Similarly to 3T3-L1 cells, the levels of UCP1 and PGC-1α proteins in hADSCs exposed to milk exosomes were also reduced by miR-11987 inhibitors compared to those in control cells (Figure 7B). We found that in the presence of milk exosomes, 3T3-L1 cells or hADSCs with miR-11987 inhibitors expressed lower levels of mitochondrial respiratory chain complex-related genes than the counterpart control cells (Figure 7C,D). Next, we analyzed the oxygen consumption rate in cells transfected with miR-11987 inhibitors. We observed that miR-11987 inhibitors led to a significant decrease in basal and maximal oxygen consumption rates in 3T3-L1 and hADSCs compared to control cells (Figure 7E,F). Together, these results suggest that miR-11987 derived from milk exosomes promotes mitochondrial activity in 3T3-L1 cells and hADSCs.

## 3. Discussion

Research on exosomes in the context of obesity has demonstrated varied effects contingent upon their cellular origin. Notably, the administration of exosomes isolated from brown adipose tissue to high-fat diet-induced obese mice has been found to mitigate body weight gain, decrease blood glucose levels, and abate lipid accumulation in the body [25]. In contrast, adipocyte-secreted exosomes promote weight gain, inflammation, and metabolic disorders [26]. The therapeutic potential of exosomes in mitigating obesity is reliant upon their cellular origin. The present investigation has demonstrated that milk exosomes originating from bovine mammary cells possess the ability to suppress high-fat diet-induced obesity in mice via the promotion of white adipose tissue browning.

Exosomes are found in foods derived from vegetables, fruits, and livestock, and plant-derived exosomes have been shown to improve obesity and diabetes. The administration of blueberry-derived exosome-like nanoparticles improved insulin resistance and attenuated the accumulation of lipid droplets in the liver of obese mice [27]. Garlic exosome-like nanoparticle-treated obese mice displayed improved glucose tolerance and insulin sensitivity [28]. In addition, the oral supplementation of ginger-derived exosomal nanoparticles prevented insulin resistance in obese mice by increasing the expression of Foxa2 [29]. Studies have established that the oral delivery of bovine milk exosomes to mice can impact the gut microbiota, fortify gut barrier integrity, and enhance immune function [30,31,32]. Bovine milk exosomes also exert protective effects against oxidative stress in intestinal crypt epithelial cells [33,34]. Milk exosomes can be absorbed in the body through the digestive system and exert their effects on various organs. Milk exosomes labeled with fluorophores accumulate in the liver, spleen, lungs, heart, kidneys, and brain in mice and pigs, although adipose tissues have not been investigated [35]. In our study, we observed that the oral administration of milk exosomes to mice for 14 weeks reduced obesity induced by a high-fat diet. These results suggest that milk exosomes absorbed into the body through the digestive system act on white fat to induce browning.

Milk-derived microRNAs may serve as a novel functional component in human health [36]. The administration of bovine and porcine milk exosomes via the oral route in piglets led to discernible differences in the microRNA profiles found in the piglet serum. Notably, the presence of miR-2284, miR-2291, miR-7134, and miR-1343 is contingent upon the exosome type administered [37]. The treatment of murine and human macrophages with bovine milk exosomes results in the detection of bovine milk exosome-derived mIR-106a, miR-451, and miR-181a in macrophage cells [38]. When human peripheral blood mononuclear cells are incubated with milk exosomes, miR-29b and miR-200c on the milk exosomes are taken up by the cells and reduce the expression of their target genes, Runx2 and Zeb [39]. In our study, the treatment of 3T3-L1 and hADSC cells with milk exosomes promoted the expression of thermogenic genes and mitochondrial activity. In addition, feeding obese mice with bovine milk exosomes including miR-11987 resulted in a reduction in body weight and fat mass. The findings of this study suggest that milk exosome-derived microRNAs are incorporated into host cells, thus serving as regulators of intracellular gene expression.

The present study reports the anti-obesity effect of miR-11987, which was previously identified as a microRNA with a pivotal role in asparanin-induced anti-cancer effects in endometrial cancer cells [40]. Currently, no additional functionalities of miR-11987 have been elucidated. Several microRNAs present in milk exosomes have been reported to play functional roles, including mIR-21, miR-92a, and miR-148. A comprehensive electronic search of the PubMed database reveals studies investigating circulating microRNAs for diagnosing obesity in humans, which report reduced levels of miR-21 in the blood of obese individuals [41]. Conversely, miR-92a demonstrates upregulated expression in states of obesity, and the inhibition of miR-92a has been shown to promote the differentiation of brown adipocytes and induce thermogenesis through its targeting of SMAD7 [42,43]. The expression levels of miR-148a are increased in adipose tissues from individuals with obesity, and this effect is mediated through the suppression of its target gene, Wnt1, which serves as an endogenous inhibitor of adipogenesis [44]. Although milk exosomes contain both miR-11987 with anti-obesity effects and miR-92a and miR-148a with pro-obesity effects, the current study suggests that the protective effect of milk exosomes against obesity is mainly attributed to the higher abundance of miR-11987 in milk exosomes, which is reported to be 3–7 times more abundant than miR-92a and miR-148a based on RNA-seq analysis. The findings indicate that the bioactivity of milk exosomes could be influenced by the composition of specific microRNAs present in these exosomes. The composition of microRNAs in milk exosomes is subject to variation, which can be attributed to the physiological state of the lactating cow responsible for milk production [45]. Therefore, further investigations are warranted to elucidate the functional roles of numerous microRNAs present in milk exosomes and to delineate the alterations in microRNA profiles within exosomes in response to various physiological conditions in lactating cows.

## 4. Materials and Methods

### 4.1. Cell Culture

Mouse 3T3-L1 pre-adipocytes were maintained in growth medium containing Dulbecco’s modified Eagle’s medium (DMEM; Hyclone, Logan, UT, USA) supplemented with 10% bovine calf serum (TCB, Long Beach, CA, USA) and 1% penicillin/streptomycin (Hyclone). 3T3-L1 pre-adipocytes were incubated in differentiation medium (DMI) to induce mature adipocytes. DMI was composed of insulin (10 μg/mL, Sigma-Aldrich, St. Louis, MO, USA), dexamethasone (1 μM, Sigma-Aldrich), and 3-isobutyl-1-methyl-xanthine (IBMX; 0.5 mM, Sigma-Aldrich) in DMEM. At 2 days after reaching confluence (day 0), cells were cultured in DMI, followed by incubation in DMEM containing 10% FBS and insulin. To induce beige adipocytes, 1 μM rosiglitazone, 50 nM T3, and 0.5 mM IBMX were added in confluent 3T3-L1 cells. Human adipose-derived stem cells (hADSCs; Lonza, Basel, Switzerland) were cultured in adipose-derived stem cell growth medium (Lonza). The cells were incubated at 37 °C in 5% CO_2_ atmosphere. To induce adipogenic differentiation, hADSCs were incubated in serum-free medium containing 10% FBS, 10 μg/mL insulin, 1 μM dexamethasone, 0.5 mM IBMX, and 100 μM indomethacin. The medium was refreshed every 3 days. 3T3-L1 cells and hADSCs were treated with milk exosomes every two days after day 0 of adipocyte differentiation.

### 4.2. Exosome Purification

Commercial milk was centrifuged at 2000× *g* for 10 min, and the supernatant was re-centrifuged at 10,000× *g* for 10 min. The collected supernatant passed through 0.45 μm and 0.2 μm filters. The filtered samples were mixed with phosphate-buffered saline (PBS) and Exoquick exosome precipitation solution (System Biosciences, Mountain View, CA, USA), and incubated for 30 min. The mixture was centrifuged at 10,000× *g* for 30 min to obtain an exosome pellet and resuspended in PBS. The size distribution of milk exosomes was measured using Zetasizer Nano ZS90 (Malvern Instruments, Orsay, France). The isolated exosomes were stored at −80 °C for further use.

### 4.3. Transmission Electron Microscopy

Exosomes were fixed with 2% glutaraldehyde for 30 min at room temperature. The fixed exosomes were loaded on 200 mesh carbon-coated formvar copper grids (GSAU200F-50, ProSciTech (Kirwan QLD, Australia)), washed with distilled water, and negatively stained with 2 % uranyl acetate. Electron micrographs were captured with a BM-UltraScan camera (Gatan, Pleasanton, CA, USA) attached to an FEI Tecnai F20 electron microscope (Hillsboro, OR, USA) operating at a 200 kV acceleration voltage.

### 4.4. Exosome Uptake Analysis

Milk exosomes were labeled by PKH26 (Sigma-Aldrich). The labeled exosomes were isolated using Exoquick exosome precipitation solution. 3T3-L1 cells and hADSCs were incubated with PKH26-labeled exosomes. Images were acquired using the fluorescence microscope.

### 4.5. Animal Experiments

Healthy male C57BL/6J mice aged 8 weeks were purchased from Raonbio Inc. (Yongin, Republic of Korea). Animal handling and procedure were conducted in accordance with ethical regulations approved by the Animal Care and Use Committee of Kyung Hee University (KHSASP-19-308). Animals were randomly allocated to a normal chow diet (CD, D12450B, Research Diets, Inc., New Brunswick, NJ, USA) or a high-fat diet (HFD, 60% of calories, D12492, Research Diets). Mice fed a high-fat diet (HFD) were gavage-administered milk exosomes or PBS every three days. The concentration of milk exosomes treatment was 50, 150, and 300 μg/mL at a dose of 200 μL. The body weight and feed intake of mice were monitored daily.

### 4.6. Transfection

3T3-L1 and ADSCs were seeded in 6-well plates before transfection. MicroRNA mimic and siRNA oligos were purchased from GenePharma (Shanghai, China). 3T3-L1 and hADSCs were transfected with 50 nM microRNA mimic or microRNA scrambles using lipofectamine 2000 reagent (Invitrogen, Carlsbad, CA, USA). To knock down the expression of Runx1t1, 3T3-L1 and hADSCs were transfected with 50 nM siRNA against Runx1t1 or negative control siRNA oligos using lipofectamine 2000. After a day of transfection, the cells were incubated in differentiation medium.

### 4.7. RNA Isolation and Quantitative Real-Time PCR (qRT-PCR)

Total RNA was extracted with Trisure reagent (Bioline, London, UK). A reverse transcription system (Promega, Madison, WI, USA) was used for cDNA synthesis, and qRT-PCR was carried out with SYBR Green PCR master mix (Thermo Fisher Sci., Waltham, MA, USA) on a Roto Gene Q thermocycler (Qiagen, Hilden, Germany). Expression levels were normalized to the internal controls (β-actin or U6 snRNA). The 2^−ΔΔCt^ method was used to evaluate the relative expression levels. RT primers and a microRNA universal TaqMan probe were purchased from Applied Biosystems (Foster City, CA, USA). The primer sequences are listed in Supplementary Table S1.

### 4.8. Western Blot Analysis

The frozen mouse tissues were homogenized. Cells and tissues were lysed with RIPA buffer (150 mM sodium chloride, 1% Trion X-100, 0.5% sodium deoxycholate, 0.1% SDS, and 50 mM Tris) and protease and phosphatase inhibitor cocktail (Roche Diagnostics Corp, Pleasanton, CA, USA). Protein concentration was quantified using Bradford Reagent (Bio-Rad, Hercules, CA, USA). Equal amounts of protein were loaded onto 8~10% SDS-PAGE, transferred to nitrocellulose membranes (Whatman international, Dassel, Germany), and blocked with 5% skim milk followed by incubating overnight at 4 °C with primary antibodies. The primary antibodies were anti-TSG 101 (Abcam, Cambridge, MA, USA), anti-CD63 (Abcam), anti-HSP70 (Invitrogen), anti-UCP1 (Abcam), anti-PGC1α (Boster Bio, Pleasanton, CA, USA), anti-NDUFB8 (Invitrogen), anti-SDHB (Invitrogen), anti-UQCRC2 (Abcam), anti-COXIV (Abcam), anti-ATP5A (Invitrogen), and anti-β-actin (Sigma). Anti-β-actin was employed as a loading control. The membranes were then incubated with the secondary antibody. Protein bands were visualized using ECL chemiluminescent reagent (Advansta Inc, Menlo Park, CA, USA).

### 4.9. Measurement of Oxygen Consumption Rate

3T3-L1 preadipocytes and hADSCs were seeded on 24 multi-well plates (Seahorse Bioscience Inc., Billerica, MA, USA). Cells differentiated into mature adipocytes in adipogenic differentiation medium were treated with milk exosomes. The cells were placed in a 37 °C incubator without CO_2_ for 1 h and were sequentially injected with oligomycin (inhibition of ATP synthase), FCCP, and antimycin A/rotenone to assess different components of oxygen consumption. Uncoupled and maximal OCR were determined using 1 μM oligomycin and 0.75 μM FCCP sequentially. To inhibit mitochondrial respiration, 1 μM antimycin A/rotenone was used. The oxygen consumption rate (OCR) of the differentiated cells was determined using a Seahorse XFe24 Extracellular Flux Analyzer (Agilent, Santa Clara, CA, USA).

### 4.10. RNA Sequencing Analysis

Total RNA was isolated from milk exosomes using TRIsure reagent (Bioline). RNA-seq analysis was performed by e-Biogen Inc. (Seoul, Republic of Korea). The quality of RNA was confirmed using 1% agarose denaturing gel and the Agilent 2100 bio-analyzer (Agilent Technologies, Palo Alto, CA, USA). The construction of libraries was performed using the NEBNext Multiplex Small RNA Library Prep kit (New England Biolabs, Ipswich, MA, USA), according to the manufacturer’s instructions. The library was sequenced on NextSeq 500 (Illumina, Inc., San Diego, CA, USA) with single-end 75 bp reads. Raw reads were collected using the Illumina analysis software. Data mining and graphic visualization were performed using ExDEGA version 2.4.5 (e-Biogen Inc., Seoul, Republic of Korea).

### 4.11. Statisti cal Analysis

Data are presented as mean ± standard error of the mean. We used Student’s t test for two-group comparisons and one-way ANOVA for more-than-two-group comparisons to calculate the *p* values. A value of *p* < 0.05 was considered statistically significant, and statistical analyses were conducted using GraphPad Prism version 8.0 (GraphPad Software, San Diego, CA, USA).

## 5. Conclusions

In conclusion, the present study highlights the therapeutic potential of milk exosomes enriched with miR-11987 for mitigating obesity by inducing the browning of white adipocytes. Moreover, the presence of bioactive microRNAs in milk exosomes shows their potential for developing novel strategies to prevent and treat obesity, warranting further investigations in this field.

## Data Availability

The data from this study are available upon reasonable request to the corresponding author.

## Supplementary Materials

The following supporting information can be downloaded at: https://www.mdpi.com/article/10.3390/ijms26136006/s1.
