# Supplementary material for: Exosome-Derived miR-11987 in Bovine Milk Inhibits Obesity Through Browning of White Fat
Source: Int J Mol Sci. 2025 Jun 23;26(13):6006. doi: 10.3390/ijms26136006 (PMC12249585; doi:10.3390/ijms26136006)
Supplement: Supplementary file 1 [file ijms-26-06006-s001.zip › ijms-3701419-supplementary author resubmit.pdf]

Supplementary Table S1. Primer sequences used for qRT-PCR

Gene	Sequence	Species
β-actin	(F) 5’-GTGACGTTGACATCCGTAAAGA-3’<br>(R) 5’-GCCGGACTCATCGTACTCC-3’	Mouse
UCP1	(F) 5'-AGGCTTCCAGTACCATTAGGT-3'<br>(R) 5'-CTGAGTGAGGCAAAGCTGATTT-3'	Mouse
TNFRSF9	(F) 5'-CGTGCAGAACTCCTGTGATAAC-3'<br>(R) 5'-GTCCACCTATGCTGGAGAAGG-3'	Mouse
CITED1	(F) 5'-AACCTTGGAGTGAAGGATCGC-3'<br>(R) 5'-GTAGGAGAGCCTATTGGAGATGT-3'	Mouse
EVA1A	(F) 5'-GGGGAGACCGAAGGAAATGAGA-3'<br>(R) 5'-CTCCAGCCCTGCACACTCTA-3'	Mouse
PDK4	(F) 5'-AGGGAGGTCGAGCTGTTCTC-3'<br>(R) 5'-GGAGTGTTCACTAAGCGGTCA-3'	Mouse
PGC-1α	(F) 5'-TATGGAGTGACATAGAGTGTGCT-3'<br>(R) 5'-CCACTTCAATCCACCCAGAAAG-3'	Mouse
TNFRSF9	(F) 5'-CGTGCAGAACTCCTGTGATAAC-3'<br>(R) 5'-GTCCACCTATGCTGGAGAAGG-3'	Mouse
EAR2	(F) 5'-GAGGACGATTCGGCGTCAC-3'<br>(R) 5'-GTAATGCTTTCCACTGGACTTGT--3'	Mouse
CD40	(F) 5'-TGTCATCTGTGAAAAGGTGGTC-3'<br>(R) 5'-ACTGGAGCAGCGGTGTTATG-3'	Mouse
EBF3	(F) 5'-TCACCCTCCCTTCAAACTGTA-3'<br>(R) 5'-GTTTCACTGCGGAGATGACAT-3'	Mouse
HSL	(F) 5'-ATGGATTTACGCACGATGACA-3'<br>(R) 5'-TAGCGTGACATACTCTTGCAG-3'	Mouse
ATGL	(F) 5'-CAACGCCACTCACATCTACGG--3'<br>(R) 5'-GGACACCTCAATAATGTTGGCAC-3'	Mouse
NDUFB8	(F) 5'-TGTTGCCGGGGTCATATCCTA-3'<br>(R) 5'-AGCATCGGGTAGTCGCCATA-3'	Mouse
SDHB	(F) 5'-AATTTGCCATTTACCGATGGGA-3'<br>(R) 5'-AGCATCCAACACCATAGGTCC-3'	Mouse
UQCRC2	(F) 5'-AAAGTTGCCCCGAAGGTTAAA-3'<br>(R) 5'-GAGCATAGTTTTCCAGAGAAGCA-3'	Mouse
COXIV	(F) 5'-ATTGGCAAGAGAGCCATTTCTAC-3'<br>(R) 5'-CACGCCGATCAGCGTAAGT-3'	Mouse
ATP5A	(F) 5'-TCTCCATGCCTCTAACACTCG-3'<br>(R) 5'-CCAGGTCAACAGACGTGTCAG-3'	Mouse
Adiponectin	(F) 5'-ACTCCTGGAGAGAAGGGAGA-3'<br>(R) 5'-GAATGGGTACATTGGGAACA-3'	Mouse
Leptin	(F) 5'-GACACCAAAACCCTCATCAAG-3'<br>(R) 5'-GCATTCAGGGCTAACATCCAA-3'	Mouse
F4/80	(F) 5'-CCCCAGTGTCCTTACAGAGTG-3'<br>(R) 5'-GTGCCCAGAGTGGATGTCT-3'	Mouse
TNF-alpha	(F) 5'-GACGTGGAACTGGCAGAAGAG-3'<br>(R) 5'-TTGGTGGTTTGTGAGTGTGAG-3'	Mouse
MCP1	(F) 5'-AGGTGTCCCAAAGAAGCTGT-3'<br>(R) 5'-AAGACCTTAGGGCAGATGCAG-3'	Mouse

IL-6	(F) 5’-ACAAGTCCGGAGAGGAGACT-3’	Mouse
	(R) 5’-TGTGACTCCAGCTTATCTCTTGG-3’	
IL-10	(F) 5'-GCTCTTGCACTACCAAAGCC-3'	Mouse
	(R) 5'-CTGCTGATCCTCATGCCAGT -3'	
Ubxn7	(F) 5'-GGTGCAAGTGAAAGTGTAGGAA-3'	Mouse
	(R) 5'-AACTGGTACTAGGCTCCTCAG-3'	
Dock5	(F) 5'-CCTGGAACCCCTGTTTGAAC-3'	Mouse
	(R) 5'-GCAATGACTTGTGATTTGCTCAG-3'	
Cfap58	(F) 5'-AAGCTACTGGCTGGCTCAAG-3'	Mouse
	(R) 5'-CTGTCCACGTGCTTCAGGTA-3'	
Runx1t1	(F) 5'-ATGCCTGATCGTACCGAGAAG-3'	Mouse
	(R) 5'-GTCGTTGGCGTAAATGAGCTG-3'	
β-actin	(F) 5'-CATGTACGTTGCTATCCAGGC-3'	Human
	(R) 5'-CTCCTTAATGTCACGCACGAT -3'	
UCP1	(F) 5'-AGGATCGGCCTCTACGACAC--3'	Human
	(R) 5'-GCCCAATGAATACTGCCACTC -3'	
PGC-1α	(F) 5'-TCTGAGTCTGTATGGAGTGACAT-3'	Human
	(R) 5'-CCAAGTCGTTCACATCTAGTTCA -3'	
TNFRSF9	(F) 5'-AGCTGTTACAACATAGTAGCCAC-3'	Human
	(R) 5'-GGACAGGGACTGCAAATCTGAT -3'	
EAR2	(F) 5'-GAGCGGCAAGCATTACGGT-3'	Human
	(R) 5'-GGCAGGTGTAGCTGAGGTT-3'	
CD40	(F) 5'-ACTGAAACGGAATGCCTTCCT-3'	Human
	(R) 5'-CCTCACTCGTACAGTGCCA-3'	
EBF3	(F) 5'-AACAGGCCATCGTCTACGAG-3'	Human
	(R) 5'-GGCGTTTCGTTTCTATTGCCA-3'	
EVA1A	(F) 5'-GCAAGACGCGAAACCTGAAC-3'	Human
	(R) 5'- TTCAAATCTGGGCTCGTCCC-3'	
NDUFB8	(F) 5'-CCGCCAAGAAGTATAATATGCGT-3'	Human
	(R) 5'-TATCCACACGGTTCCTGTTGT- -3'	
SDHB	(F) 5'-GACACCAACCTCAATAAGGTCTC-3'	Human
	(R) 5'-GGCTCAATGGATTTGTACTGTGC -3'	
UQCRC2	(F) 5'-TTCAGCAATTTAGGAACCACCC-3'	Human
	(R) 5'-GGTCACACTTAATTTGCCACCAA -3'	
COXIV	(F) 5'-CTTCAGCAGGAAGCACCGAAT-3'	Human
	(R) 5'- AGAGGTGGAAATTGCTCGCT-3'	
ATP5A	(F) 5'-AAAGACTGGGACTGCTGAGA-3'	Human
	(R) 5'- GGCATCAACTACACGACCCA-3'	
Runx1t1	(F) 5'-ATGCCAGACTCACCTGTGGAT-3'	Human
	(R) 5'-GGCTGTAGGAGAATGGCTCG -3'	

Supplementary Table S2. Read count of microRNA in milk exosomes using RNA-seq analysis

MicroRNA	Read count (>500)
**miR-11987**	**7697**
**miR-122a**	**4913**
**miR-11980**	**4728**
**miR-21-5p**	**3957**
**miR-1777b**	**2588**
**miR-2478**	**2570**
**miR-92a**	**2563**
**miR-1777a**	**2505**
**miR-2430**	**2036**
**miR-2305**	**1551**
**let-7f**	**1330**
**miR-2412**	**1140**
**miR-148a**	**887**
