# Supplementary material for: Exosome-Derived miR-11987 in Bovine Milk Inhibits Obesity Through Browning of White Fat
Source: Int J Mol Sci. 2025 Jun 23;26(13):6006. doi: 10.3390/ijms26136006 (PMC12249585; doi:10.3390/ijms26136006)
Supplement: Supplementary file 1 [file ijms-26-06006-s001.zip › ijms-3701419-WB.pdf]

Figure 1.

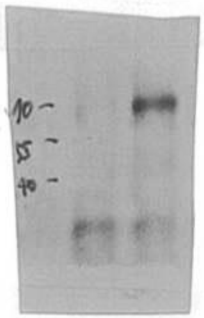

HSP70

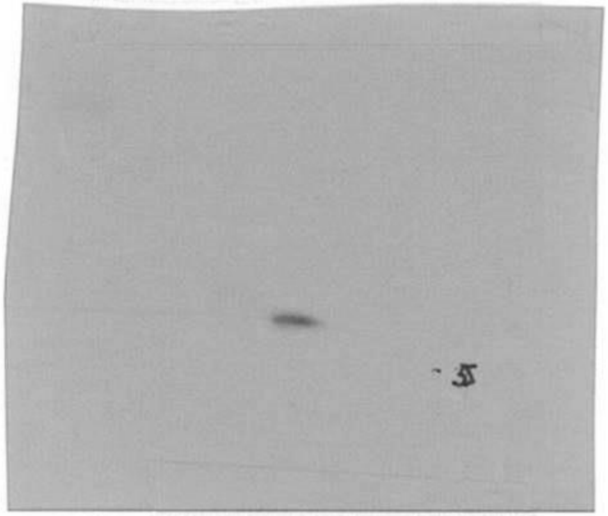

CD63

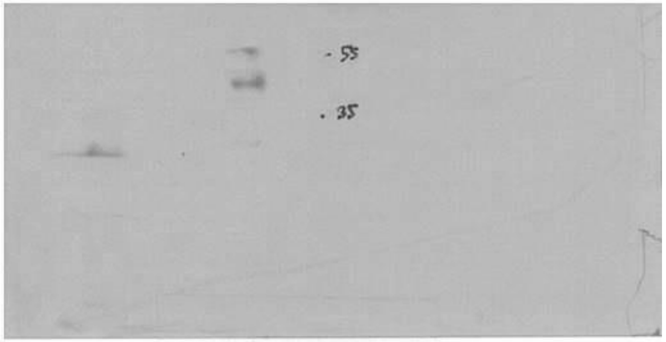

TSG101

Figure 2.

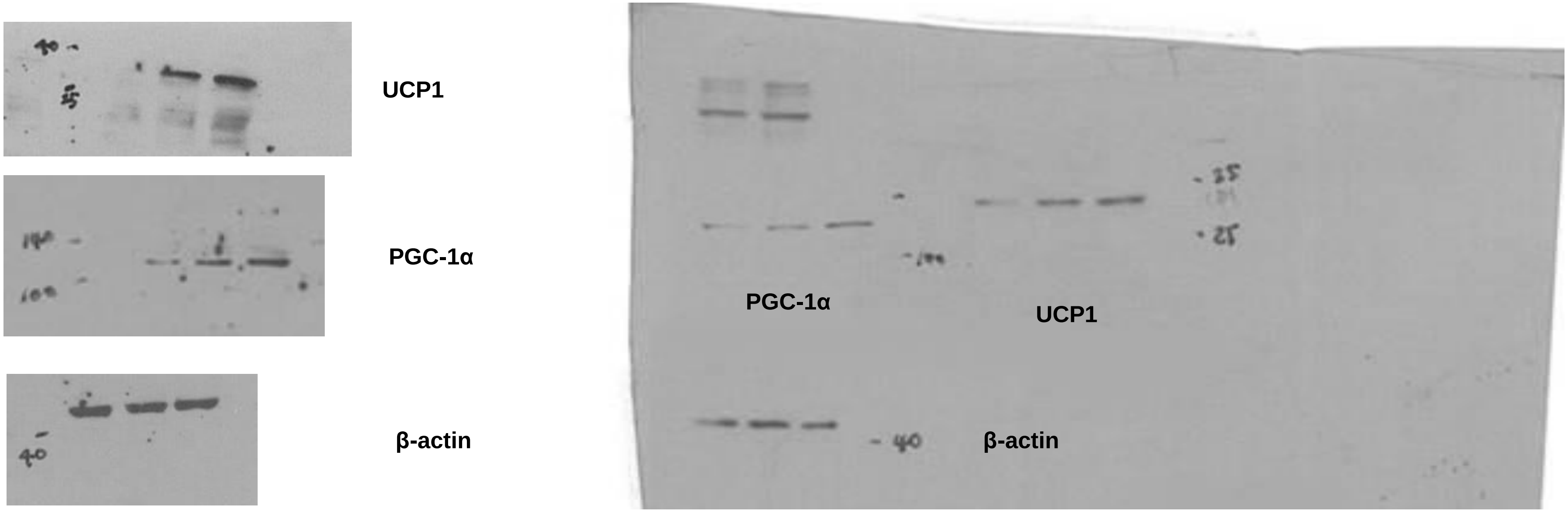

Figure 3.

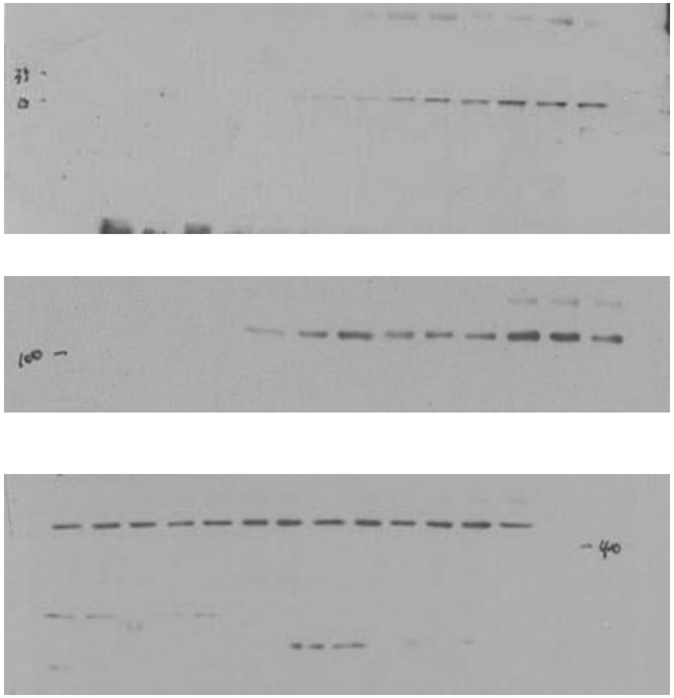

UCP1

PGC-1α

β-actin

Figure 4.

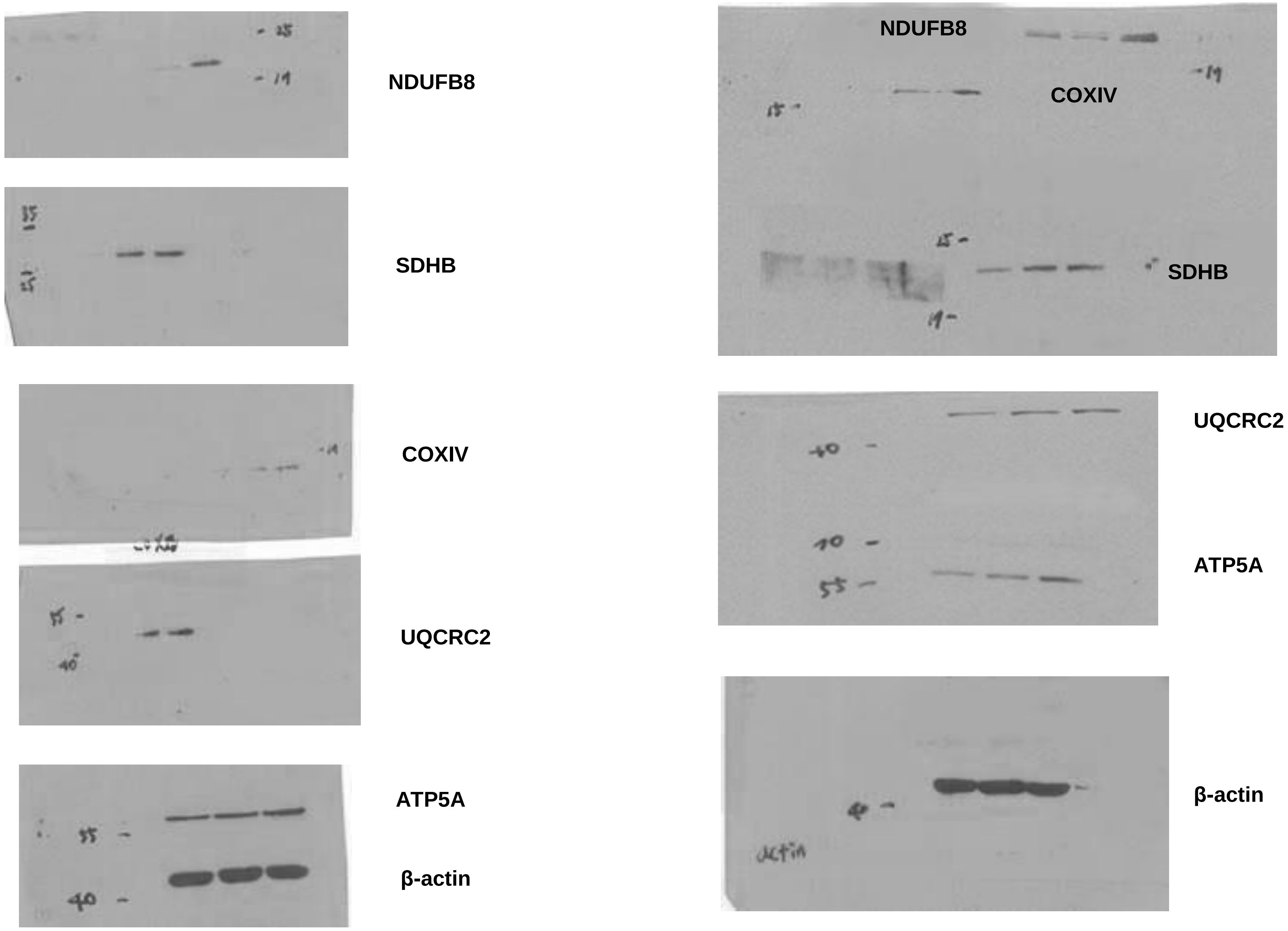
NDUFB8
SDHB
COXIV
UQCRC2
ATP5A
β-actin
NDUFB8
COXIV
SDHB
UQCRC2
ATP5A
β-actin

Figure 4.

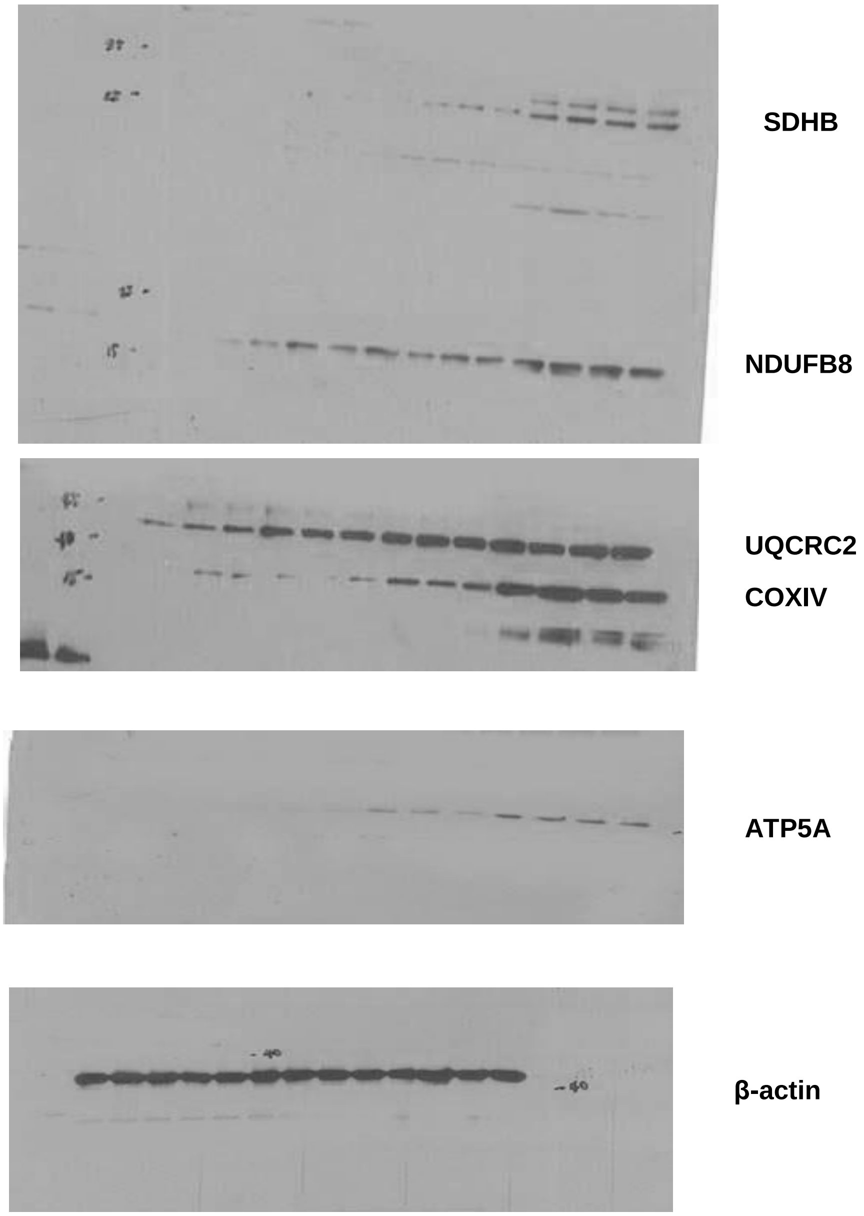

Figure 5.

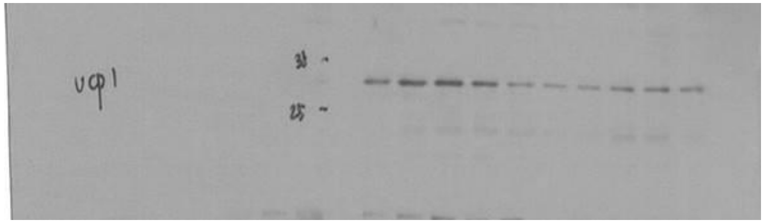

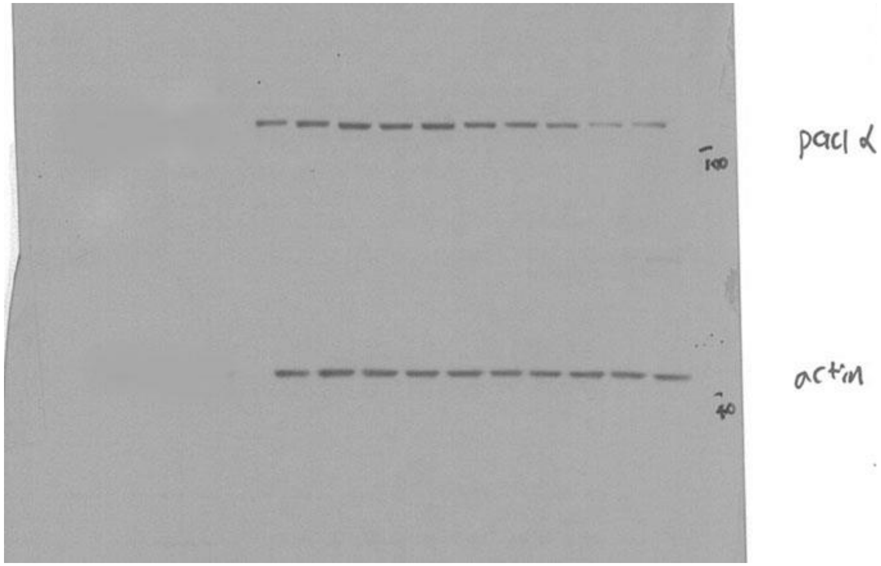

Figure 6.

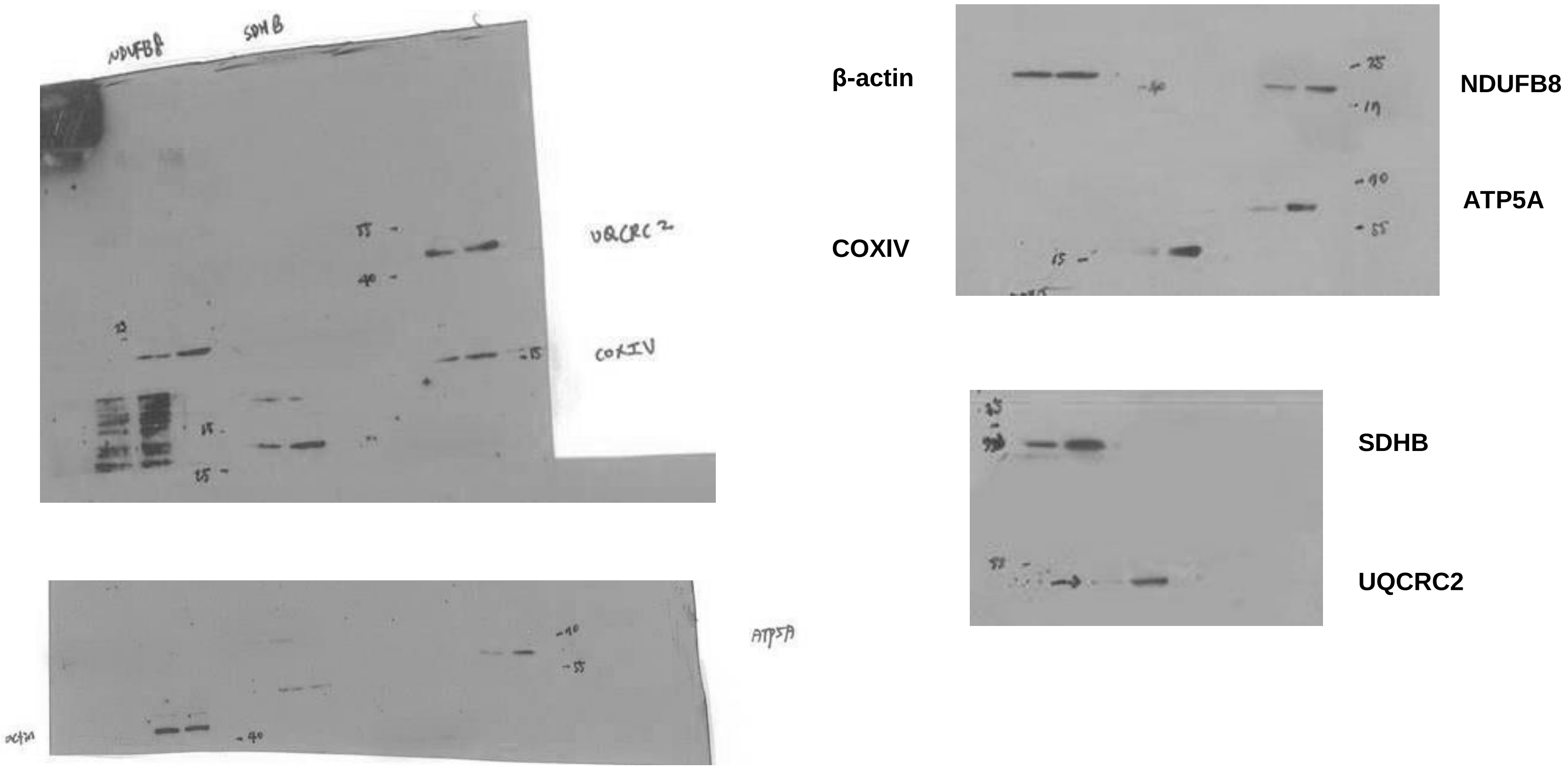

Figure 7.

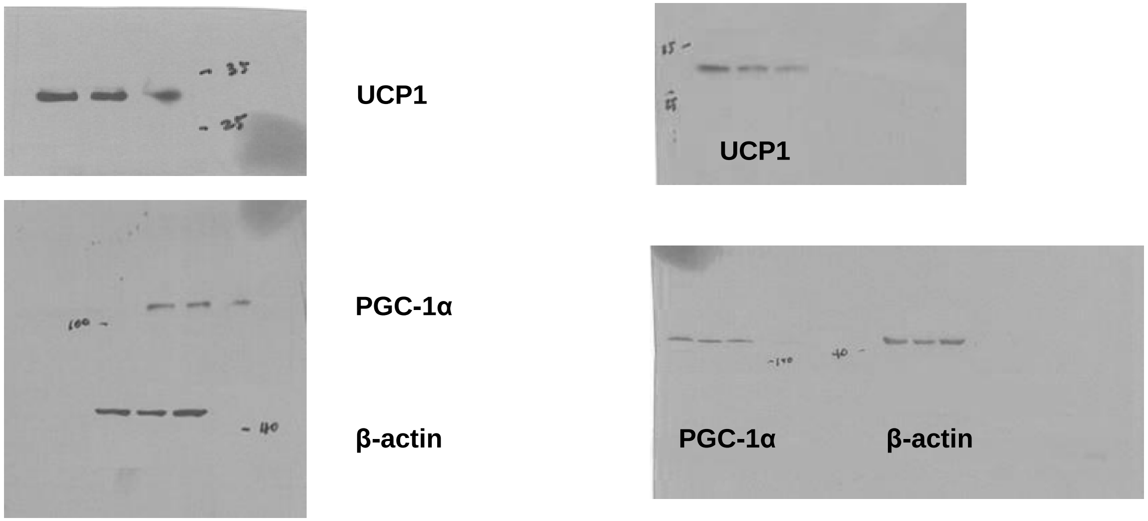

Figure 7.

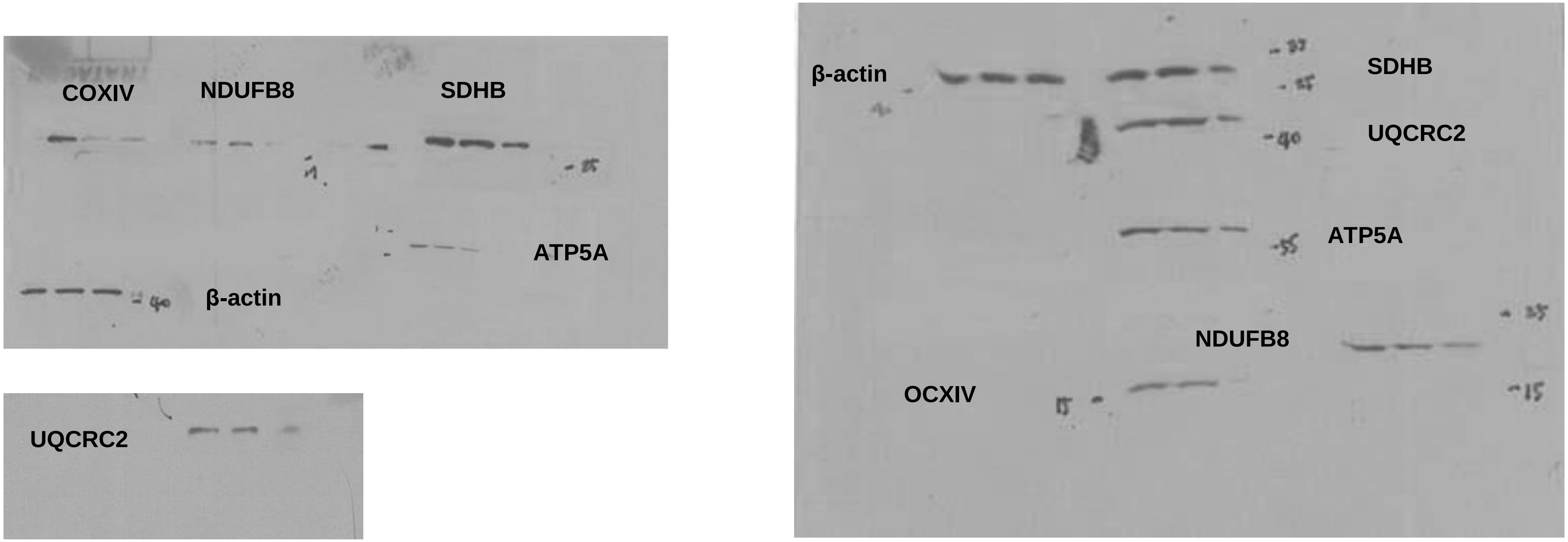
COXIV
NDUFB8
SDHB
ATP5A
β-actin
UQCRC2
β-actin
SDHB
UQCRC2
ATP5A
NDUFB8
OCXIV
